# Impact of the COVID-19 pandemic and post-epidemic periods on the process of endovascular treatment for acute anterior circulation ischaemic stroke

**DOI:** 10.1186/s12883-021-02262-0

**Published:** 2021-06-24

**Authors:** Tangqin Zhang, Chu Chen, Xiangjun Xu, Junfeng Xu, Ke Yang, Youqing Xu, Lili Yuan, Qian Yang, Xianjun Huang, Zhiming Zhou

**Affiliations:** Department of Neurology, Yijishan Hospital, Wannan Medical College, 2# Zheshan West Road, Wuhu, 241001 Anhui Province China

**Keywords:** Acute ischaemic stroke, Endovascular treatment, COVID-19, Pandemic, Post-epidemic

## Abstract

**Background and purpose:**

The purpose of our study was to analyse endovascular treatment (EVT) in patients presenting acute anterior circulation ischemic stroke with large-vessel occlusion (AIS-LVO) during the pandemic and post-epidemic periods.

**Methods:**

Patients with AIS-LVO of the anterior circulation who underwent EVT were enrolled. According to the times of Wuhan closure and reopening, patients were divided into a pre-pandemic group (from November 8, 2019, to January 22, 2020), pandemic group (from January 23, 2020, to April 8, 2020) and post-epidemic group (from April 9, 2020, to June 24, 2020). The primary endpoints were the time delay among symptom onset to arriving hospital door, to groining puncture and to vascular reperfusion. Secondary endpoints were the functional outcomes evaluated by 90-day modified Rankin scale (mRS) score.

**Results:**

In total, the times from onset to reperfusion (OTR, median 356 min vs. 310 min, *p* = 0.041) and onset to door (OTD, median 238 min vs. 167 min, *p* = 0.017) were prolonged in the pandemic group compared to the pre-pandemic group, and the delay continue in the post-epidemic period. In the subgroup analysis, the time from door to imaging (DTI) was significantly prolonged during the pandemic period. Interestingly, the prolonged DTI was corrected in the directly admitted subgroup during post-epidemic period. In addition, the functional outcomes showed no significant differences across the three periods.

**Conclusions:**

Total time and prehospital time were prolonged during the pandemic and post-epidemic periods. Urgent public education and improved in-hospital screening processes are necessary to decrease time delays.

## Introduction

Since the first case of the novel coronavirus disease 2019 (COVID-19) patient was reported in Wuhan, China in December 2019, the COVID-19 has become a global pandemic [1, 2]. To control the spread of the disease, Wuhan city was placed on lockdown on January 23, 2020, and the government has implemented several anti-epidemic measures, for example people were required decreasing contact and keeping social distance, the floating population need to measure body temperature at free-way exit and entrance, the patients were required accepting strict COVID-19 screening before admitted [3, 4].

During the pandemic, a few studies reported that the number of acute anterior circulation ischemic stroke with large-vessel occlusion (AIS-LVO) patients who underwent endovascular treatment (EVT) decreased and the time from stroke onset to puncture (OTP) was significantly prolonged [5–12]. Fortunately, with the reopening of Wuhan on April 8, 2020, it means the domestic epidemic has been effectively controlled. However, prehospital screening is still strictly enforced to prevent retransmission of the outbreak. The standardization of outbreak prevention measures may complicate the medical process, which may lead to delays in AIS-LVO patients.

Therefore, we conducted a retrospective study of AIS-LVO patients who received EVT at our hospital from the pre-pandemic to the post-epidemic period. Aimed to investigate the impact of the COVID-19 pandemic and post-epidemic periods on the process and outcome of EVT for AIS-LVO patients.

## Methods

### Patient selection

We continuously included all AIS-LVO patients with anterior circulation who underwent EVT in Yijishan Hospital of Wannan Medical College from January 23, 2020, to April 8, 2020 (76 days from the day Wuhan closure to reopening), constituting the pandemic group. AIS-LVO patients ranged from another 76 days after Wuhan’s reopening (from April 9, 2020, to June 24, 2020), constituted the post-epidemic group. AIS-LVO patients before the pandemic of November 8, 2019, to January 22, 2020 (76 days), constituted the pre-pandemic group. Patients with posterior circulation occlusion were excluded from the study. The study was approved by the Ethics Committee of the First Affiliated Hospital of Wannan Medical College (201,900,039). All methods were performed in accordance with the relevant guidelines and regulations.

### Data collection

Baseline patients’ data were collected prospectively and sequentially, including demographic characteristics (age, sex), vascular risk factors (hypertension, diabetes, atrial fibrillation), National Institute of Health Stroke Scale (NIHSS) score, Alberta Stroke Program Early CT (ASPECT) score, Trial of Org 10 172 in acute stroke treatment (TOAST), site of occlusion, rate of intravenous thrombolysis and non-eyewitness stroke. When the time of symptom onset was unknown, the last normal time of the patient was regarded as the time of occurrence of the symptom.

We collected the following time duration in different periods respectively, including (1) total time: the time from stroke onset to vascular reperfusion (OTR); (2) prehospital time: the time from stroke onset to our hospital door (OTD); (3) preparation time: the time from arriving the door of our hospital to groin puncture (DTP); and (4) procedural time: the time from puncture to reperfusion (PTR).

All patients were divided into two subgroups: directly admitted subgroup and transferred from other hospitals subgroup. The time data of two subgroups were collected separately. In the directly admitted subgroup, the time data included stroke onset to comprehensive stroke centre (CSC) door, CSC door to imaging (DTI), imaging to puncture (ITP), and puncture to reperfusion (PTR). In patients who were transferred from other institutions, the time data included stroke onset to primary stroke centre (PSC) door, PSC door to departure (door-in-door-out, DIDO), PSC door to imaging, imaging to departure, departure to CSC, CSC to puncture, and puncture to reperfusion (PTR).

Modified Rankin scale (mRS) score were obtained by telephone follow-up on the 90-day after EVT of each patient by 2 experienced interventional neurologists. The good functional outcome was defined as mRS score 0–2. Symptomatic intracranial haemorrhage (sICH) was defined as blood at any site in the brain on the CT scan, documentation by the investigator of clinical deterioration, or adverse events indicating clinical worsening or causing a decrease in the NIHSS score of 4 or more points [13].

### Statistical analysis

Continuous variables were evaluated by a histogram and the Shapiro–Wilk test, in which normally distributed continuous variables are presented as the mean ± standard deviation. Additionally, non-normally distributed continuous variables are presented as the median and interquartile range. Categorical variables are reported as frequencies and percentages. The independent T test was used for normally distributed continuous variables, and the Mann–Whitney U test was used for non-normally distributed continuous variables comparing two groups. The chi-square test or Fisher’s test was used for categorical variables. Differences were considered statistically significant at *p* < 0.05 (two-side). Data analyses were performed with SPSS version 26.0 (IBM Corp, Armonk, NY).

## Results

### Overall characteristics

**Fig. 1:**
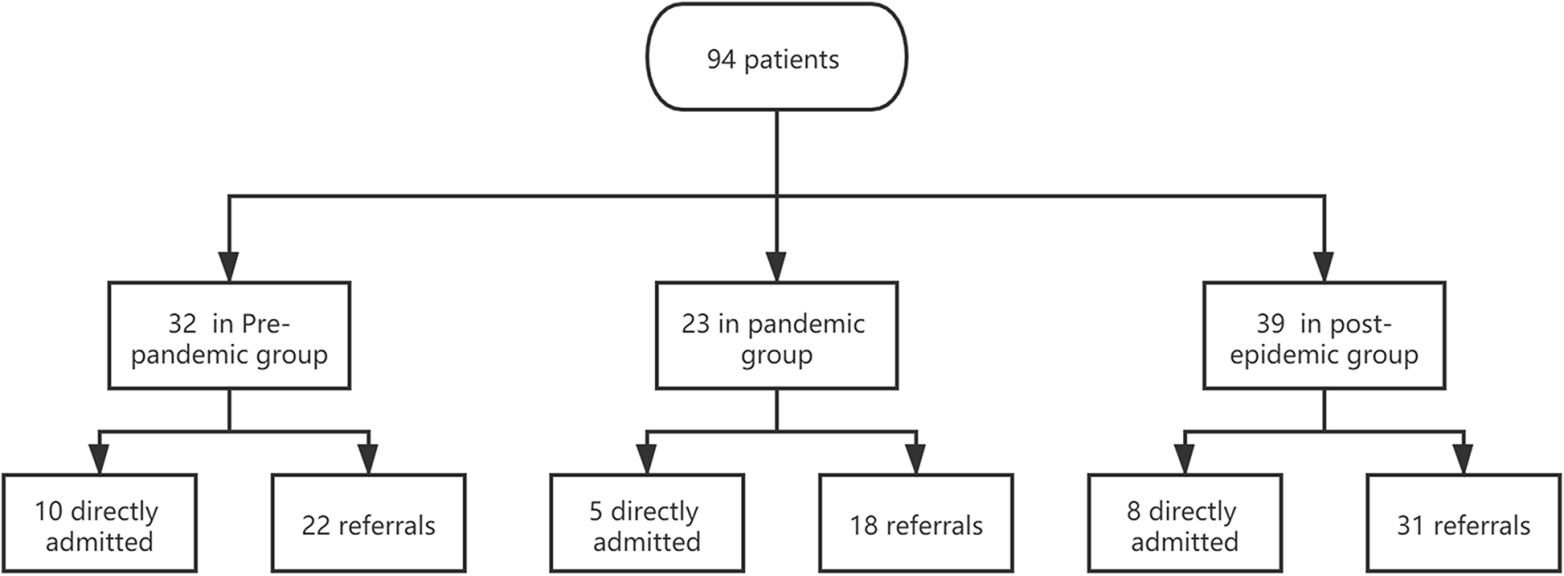
Flowchart describing the grouping of patients. We analysed 94 acute anterior circulation large vascular occlusion stroke patients who underwent endovascular treatment. According to the time of Wuhan closure and reopening, the patients were divided into the pre-pandemic group (*n* = 32), pandemic group (*n* = 23) and post-epidemic group (*n* = 39). Then, each group was divided into two subgroups according to whether patients arrived directly at our hospital

In total, 94 patients with AIS-LVO (the mean age was 68.6 ± 10.4 years, and 58.5% were men) received EVT. There were 32 patients (34.0%) in the pre-pandemic group, 23 patients (24.5%) in the pandemic group, and 39 (41.5%) patients in the post-epidemic group. The median baseline NIHSS score was 13 (IQR 10–17), and the median ASPECT score was 9 (IQR 8–10). The median total time (OTR) was 347 (IQR 263–415) min, median prehospital time (OTD) was 220 (IQR 160–290) min, median preparation time (DTP) was 60 (IQR 36–89) min. There were 71 (75.5%) patients transferred from other institutions, and 15 (16.1%) patients had unwitnessed stroke (Table 1, Fig. 1).

**Table 1:** Baseline characteristics of the study population

	Pre-pandemic group(*n* = 32)	Pandemic group(*n* = 23)	Post-epidemic group(*n* = 39)	P* value	P** value	P*** value
Age (years), mean (SD)	65.5(10.7)	70.7(10.0)	69.9(10.1)	0.077	0.079	0.784
Male gender, n (%)	21(65.6)	15(65.2)	19(48.7)	0.975	0.153	0.207
Past history, n (%)						
Hypertension	23(71.9)	10(43.5)	29(74.4)	0.034	0.814	0.015
Diabetes	4(12.5)	4(17.4)	4(10.3)	0.707	0.999	0.454
Atrial fibrillation	17(53.1)	14(60.9)	23(59.0)	0.568	0.621	0.883
Stroke aetiology, n (%)				0.984	0.854	0.981
Large vessel atherosclerosis	6(18.7)	5(21.7)	9(23.1)			
Cardioembolic	21(65.6)	15(65.2)	26(66.7)			
Others	2(6.3)	1(4.4)	1(2.6)			
Undetermined	3(9.4)	2(8.7)	3(7.7)			
Thrombus location, n (%)				0.145	0.012	0.061
Intracranial ICA	10(31.2)	5(22.7)	11(28.2)			
M1 MCA segment	10(31.2)	13(59.1)	25(64.1)			
M2 MCA segment	6(18.8)	4(18.2)	0			
ACA segment	2(6.3)	0	1(2.6)			
Tandem occlusion	4(12.5)	0	2(5.1)			
Unwitnessed onset, n (%)	3(9.4)	4(17.4)	8(21.1)	0.435	0.181	0.999
Baseline NIHSS, median (IQR)	12(9,16)	12(10,18)	14(11,18)	0.693	0.643	0.977
ASPECTS, median (IQR)	9(8,10)	8(6,9)	9(7,10)	0.141	0.651	0.077
Use of rtPA, n (%)	7(21.9)	2(8.7)	1(2.6)	0.277	0.019	0.549
Transfer from another institution, n (%)	22(68.8)	18(78.3)	31(79.5)	0.435	0.301	0.999
Total time (OTR), median (IQR)	310(254,349)	356(263,404)	366(328,507)	0.041	0.003	0.494
Prehospital time (OTD), median (IQR)	167(120,213)	238 (194,303)	244(211,339)	0.017	< 0.001	0.418
Preparation time (DTP), median (IQR)	72(41,99)	43(31,89)	60(42,80)	0.129	0.466	0.147
Procedural time (PTR), median (IQR)	47(35,68)	60(40,77)	50(40,85)	0.130	0.217	0.749
Onset to puncture (OTP), median (IQR)	240(212,300)	300(225,340)	312(260,400)	0.119	0.003	0.365

P* Pre-pandemic group vs. Pandemic group, P** Pre-pandemic group vs. Post-epidemic group,

P*** Pandemic group vs. Post-epidemic group,

*NIHSS* National Institutes of Health Stroke scale, *ASPECTS* Alberta Stroke Program Early CT score, *MCA* middle cerebral artery, *ICA* internal carotid artery, *ACA* anterior cerebral artery, *CSC* comprehensive stroke centre, *IQR* Interquartile range (25%–75%)

The pandemic group had a lower rate of hypertension than the pre-pandemic group (43.5% vs. 71.9%, *p* = 0.034) and post-epidemic group (43.5% vs. 74.4%, *p* = 0.015). The post-epidemic group had a lower rate of MCA-M2 occlusion (0 vs. 18.8%, *p* = 0.012) and receiving rtPA (2.6% vs. 21.9, *p* = 0.019) than the pre-pandemic group. There was no significant difference in other baseline data among the three groups (Table 1).

### Time delay on OTR, OTD, DTP and PTR

**Fig. 2:**
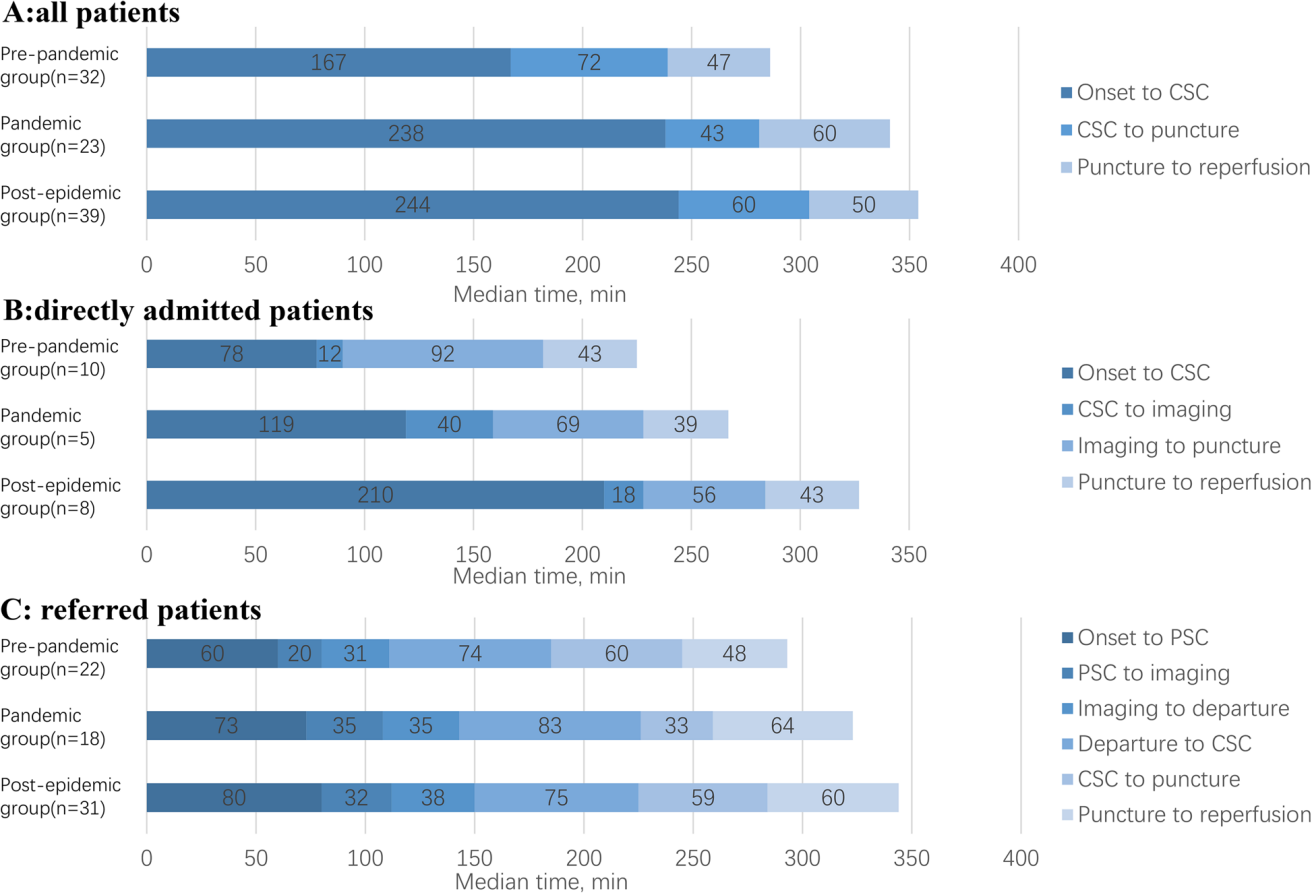
Time comparison of different groups. **A** The median time from stroke onset to reperfusion of all patients. **B** The median time from stroke onset to reperfusion of directly admitted patients. **C** The median time from stroke onset to reperfusion of transferred patients

Median total time (OTR, median 366 min) and median prehospital time (OTD, median 244 min) were the longest in the post-epidemic group. Median total time (OTR, median 356 min vs. 310 min, *p* = 0.041) and prehospital time (OTD, median 238 min vs. 167 min, *p* = 0.017) were longer in the pandemic group compared with pre-pandemic group. Median preparation time (DTP, median 43 min) was shortest in the pandemic group, although the difference was not statistically significant among three group. In addition, no significant difference in procedural time (PTR) was found across the three groups (Table 1, Fig. 2A).

In the subgroup of patients who were directly admitted (Table 2, Fig. 2B), Median total time (OTR, median 305 min) and median prehospital time (OTD, median 210 min) were also the longest in the post-epidemic group. In terms of preparation time (DTP), the median time from CSC door to imaging (DTI, median 40 min) was the longest in the pandemic group, and the median time from imaging to puncture tended to be shorter in the pandemic group (ITP, median 69 min vs. 92 min, *p* = 0.111) and post-epidemic group (ITP, median 56 min vs. 92 min, *p* = 0.248) than pre-pandemic group, although no significant difference. In addition, procedural time (PTR) was not significantly different among the three groups.

**Table 2:** Peri-procedural times of the study population

	Pre-pandemic group(*n* = 32)	Pandemic group(*n* = 23)	Post-epidemic group(*n* = 39)	P* value	P** value	P*** value
Time data for directly admitted, median (IQR)						
Total time (OTR)	260(220,330)	248(217,263)	305(115,394)	0.999	0.722	0.999
Prehospital time (OTD)	78(57,138)	119(112,234)	210(165,247)	0.330	0.096	0.624
Preparation time (DTP)	115(97,144)	93(89,109)	78(50,187)	0.391	0.594	0.661
CSC door to imaging	12(10,31)	40(24,45)	18(15,24)	0.043	0.213	0.079
Imaging to puncture	92(71,124)	69(44,73)	56(29,111)	0.111	0.248	0.770
Procedural time (PTR)	43(37,70)	39(37,40)	43(34,60)	0.240	0.755	0.556
Onset to puncture (OTP)	212(180,260)	209(180,227)	260(78,353)	0.902	0.563	0.884
Time data for referred patients, median (IQR)						
Total time (OTR)	323(263,360)	380(342,404)	379(345,518)	0.022	0.005	0.609
Prehospital time (OTD)	199(160,240)	257(209,303)	252(220,361)	0.050	0.006	0.561
Onset to PSC door	60(30,80)	73(60,120)	80(60,210)	0.037	0.007	0.677
PSC door to imaging	20(13,60)	35(16,51)	32(20,50)	0.470	0.329	0.975
Imaging to departure	31(20,50)	35(24,59)	38(16,70)	0.471	0.492	0.868
DIDO	56(35,107)	72(60,100)	67(60,100)	0.294	0.281	0.983
Departure to CSC door	74(63,90)	83(70,93)	75(60,90)	0.369	0.899	0.294
Preparation time (DTP)	60(37,80)	33(29,54)	59(42,78)	0.097	0.993	0.020
Procedural time (PTR)	48(35,66)	64(56,80)	60(40,87)	0.029	0.117	0.468
Onset to puncture (OTP)	270(225,300)	309(249,340)	313(290,420)	0.126	0.010	0.441

P* Pre-pandemic group vs. Pandemic group, P** Pre-pandemic group vs. Post-epidemic group,

P*** *P*andemic group vs. Post-epidemic group, *CSC* Comprehensive stroke centre,

*PSC* Primary stroke centre, *DIDO* Door-in-door-out, *IQR* Interquartile range (25%–75%)

In the subgroup of patients transferred from other institutions (Table 2, Fig. 2C), similar to the whole cohort, the total time (OTR) and the time from stroke onset to PCS door were significantly prolonged in the pandemic group and post-epidemic group, compared with the pre-pandemic group. There was no significant difference of median DIDO time among three group. The median CSC’s preparation time (DTP, median 33 min) was the shortest in pandemic group. Regarding procedural time, the pandemic group (PTR, median 64 min vs. 48 min, *p* = 0.029) and post-epidemic group (PTR, median 60 min vs. 48 min, *p* = 0.117) were slightly more prolonged than the pre-pandemic group.

### Outcomes of EVT patients

The reperfusion rate of the post-epidemic group tended to be higher than that of the pandemic group (92.3% vs. 73.9%, *p* = 0.066) and pre-pandemic group (92.3% vs. 78.1%, *p* = 0.168), although no significant difference. The rates of 90-day good outcome, 90-day death and sICH were similar among the three groups (Table 3).

**Table 3:** Outcomes of the study population

	Pre-pandemic group(*n* = 32)	Pandemic group(*n* = 23)	Post-epidemic group(*n* = 39)	P* value	P** value	P*** value
sICH, n (%)	3(9.4)	4(17.4)	3(7.7)	0.435	0.999	0.408
Reperfusion mTICI 2b/3	25(78.1)	17(73.9)	36(92.3)	0.717	0.168	0.066
90-day mRS ≤ 2, n (%)	18(56.3)	14(60.9)	24(61.5)	0.732	0.652	0.958
90-day mRS, median (IQR)	2(0,4)	2(1,5)	2(1,4)	0.650	0.612	0.894
90-day mortality, n (%)	6(18.8)	5(21.7)	8(20.5)	0.999	0.853	0.999

P* Pre-pandemic group vs. Pandemic group, P** Pre-pandemic group vs. Post-epidemic group,

P*** Pandemic group vs. Post-epidemic group,

*NIHSS* National Institutes of Health Stroke scale, *sICH* Symptomatic intracranial haemorrhage transformation, *mTICI* Modified Treatment in Cerebral Infarction, *mRS* Modified Rankin Scale, *IQR* Interquartile range (25%–75%)

## Discussion

In the present study, we compared in detail EVT procedures and clinical outcomes ranging from pre-pandemic period to post-epidemic period. The key findings included the following: (1) in total patients, total time (OTR) and prehospital time (OTD) were significantly prolonged during the pandemic and post-epidemic period. However, preparation time (DTP) was shortened during the pandemic period, although there is no statistical difference; (2) in the subgroup analysis, the time from door to imaging (DTI) was significantly prolonged in the directly admitted CSC subgroup and referral subgroup during the pandemic period. Interestingly, DTI was corrected in the directly admitted CSC subgroup during the post-epidemic period; (3) we did not find a delay in procedural time (PTR) in the overall analysis or the subgroup analysis; and (4) we did not find differences in the clinical outcomes among the periods.

Our study results showed that the total time (OTR) was prolonged in the pandemic period, which was consistent with previous studies [5–12]. Moreover, the delay continued in the post-epidemic period. Notably, the total time delay mainly occurred in the prehospital period. Three reasons could explain the delay. First, the reduction in person-to-person contacts as a result of the promulgation of the anti-epidemic measures, which may lead to a delayed stroke recognition [14, 15]. Another reason is the overload of Emergency Medical Dispatch (EMS) calls had led the saturation of ambulances, patients’ family members were required to drive through the temperature measurement centre at free-way exit and entrance to the hospital by themselves, it may increase pre-hospital transfer time [6, 9, 16]. Finally, the reason might be that some patients arrive at the hospital delayed due to excessive worry about being infected by the new coronavirus [17]. Therefore, we suggest that local policymakers should widely disseminate knowledge of COVID-19 and set up special emergency routes at free-way exit and entrance to reduce prehospital time by removing patients’ fear of COVID-19 and reducing the waiting time on highway stations.

Other finding from this report included the number of patients receiving intravenous thrombolysis decreased due to miss the time window during the pandemic and post-epidemic. It was associated not only with prolonged prehospital time (OTD), but also with the time delays from door to imaging (DTI). All newly admitted patients had to accept strict COVID-19 screening in the pandemic period and post-epidemic period, including answering questions about their travel history, potential contact with a confirmed case, respiratory symptoms and chest CT [9]. There was no doubt that increased procedures will lead to time delays. Therefore, DTI time was delayed in the pandemic group compared with the pre-pandemic group, regardless of PSCs and CSC. Another thing worth noting is that DTI time was corrected in CSC but was still maintained in PSCs during the post-epidemic period. This may reflect the validity of the change in the screening mode in our stroke centre, in which patients were transferred to the imaging centre simultaneously to undergoing COVID-19 screening during the post-epidemic period. Therefore, stroke centres should establish a similar process for stroke patients, in which the patient is taken directly to the imaging centre if the likelihood of AIS-LVO is high, with COVID-19 screening and preliminary physician–patient communication carried out simultaneously. In addition, PSC's COVID-19 screening results were valid in CSC, which meant that the change in screening mode can be widely promoted.

Whereas we found the time from door to imaging (DTI) was delayed, preparation time (DTP) was shortened during the pandemic period, which may be related to the decrease of the time from imaging to puncture (ITP). In CSC, referral patients’ DTP and directly admitted patients’ imaging to puncture time (ITP) decreased significantly in the pandemic period compared with the pre-pandemic period. Perhaps the primary reason is that, in CSC, the number of stroke patients decreased during the pandemic period, stroke teams had more sufficient preparation to deal with AIS-LVO patients who underwent EVT, and the catheter room was relatively idle. However, due to the number of EVT patients increased, the decreasing trend of DTP did not continue in post-epidemic period. In addition, the time from imaging to departure, departure to CSC and DIDO did not significantly change in PSC. It is worth noting that due to the relatively inconvenient transportation during the pandemic period, most patients chose to visit PSC, rather than be directly admitted to CSC. Therefore, under the background of the normalization of epidemic prevention, PSC quickly identified and transferred AIS-LVO patients, and it might be more effective to improve the process of AIS-LVO patients.

In term of procedural time (PTR), we seem to have found a slight delay in procedural time during the pandemic and post-epidemic periods, although no statistical difference. The prolonged trend is mainly seen in the referral group, which may be related to the greater delay of OTP [18, 19]. Previous large studies have shown that OTP delays increase the difficulty of successful reperfusion, which means longer procedural times and poorer outcomes [18, 19]. Therefore, physicians and local policymakers should make efforts to reduce prehospital and preparation time to improve the rate of successful reperfusion and clinical outcomes.

There is no doubt that as the domestic epidemic has been effectively controlled, the fear of the new coronavirus has largely dissipated. However, the status of EVT in AIS-LVO patients was still not satisfactory during the post-epidemic period which not only the total time (OTR), prehospital time (OTD) and preparation time (DTP) were significantly prolonged, but the number of receiving thrombolytic therapy was also significantly reduced. We concerned that continued existence of epidemic prevention measures and the significantly increased number of EVT patients were placing an enormous burden on the stroke centres health system, and that medical resources no longer meet the needs of patients. Therefore, it is urgent to simplify in-hospital screening in stroke centres.

Although the time has been prolonged during the pandemic and post-epidemic periods, there were no significant differences in all clinical outcomes, including the rate of successful reperfusion, 90-day good outcome, 90-day death and sICH. This may be related to our small sample size being insufficient to detect the outcome changes caused by time delay.

### Limitations

Our report has some limitations. One of the major limitations is the retrospective, observational design. In addition, this was a single-centre study with a small sample size. Finally, the results observed may not be applicable to other regions or countries with different geographical specificities, healthcare systems and policies for the COVID-19 pandemic. Despite the above shortcomings, this report provides important information about the time and outcome data during the pandemic period and post-epidemic period.

## Conclusions

During the COVID-19 pandemic period, total time (OTR), prehospital time (OTD) and door to imaging (DTI) were significantly prolonged. Due to the change in screening mode, the prolonged DTI was corrected in post-epidemic period. However, the total time and pre-hospital time were still prolonged. Therefore, we recommend that physicians and local policymakers pay more attention to widely disseminating knowledge of COVID-19, prehospital transfer and improvement of the in-hospital screening process to reduce the time delay during the pandemic and post-epidemic periods.

## Data Availability

The data used to support the findings of this study are available from the corresponding author upon request.
